# Application of microbiological assay to determine pharmaceutical equivalence of generic intravenous antibiotics

**DOI:** 10.1186/1472-6904-9-1

**Published:** 2009-01-16

**Authors:** Andres F Zuluaga, Maria Agudelo, Carlos A Rodriguez, Omar Vesga

**Affiliations:** 1 GRIPE: Grupo Investigador de Problemas en Enfermedades Infecciosas, University of Antioquia, Medellín, Colombia; 2 Department of Pharmacology and Toxicology, University of Antioquia, Medellin, Colombia; 3 Section of Infectious Diseases, Department of Internal Medicine, University of Antioquia, Medellin, Colombia

## Abstract

**Background:**

Demonstration of equivalent amounts of the same active pharmaceutical ingredient (API) between generic and innovator products (pharmaceutical equivalence) is a basic requirement of regulatory agencies for intravenous generic drugs prior to clinical use, and constitutes the pivotal point to assume therapeutic equivalence. Physicochemical methods are preferred instead of biological assays to determine concentration of drugs in biological fluids, but it does not permit direct quantification of potency. Here, we report a microbiological assay using large plates designed to determine potency and concentration of pharmaceutical-grade antibiotics for injection and a statistical method to assess the in vitro equivalence of generic products with respect to the innovator.

**Methods:**

The assay is based on the concentration-dependent variation of the inhibitory effect of antibiotics on reference bacteria (*B. subtilis *ATCC 6633, *S. aureus *ATCC 6538p and *S. epidermidis *ATCC 12228) in a seeded agar (Difco™ Antibiotic Media), producing a concentration-response linear relationship with two parameters: y-intercept (concentration) and slope (potency). We compared the parameters of 22 generic products (amikacin 4, gentamicin 15, and vancomycin 3 products) against the innovator and the reference powder by Overall Test for Coincidence of the Regression Lines (Graphpad Prism 5.0).

**Results:**

The validation method yielded excellent results for linearity (r^2 ^≥ 0.98), precision (intra-assay variation ≤ 11%; inter-assay variation ≤ 10%), accuracy, and specificity tests according to international pharmacopoeial requirements. Except for one generic of vancomycin that had 25% more API (P_*y*-*intercept *_= 0.001), the pharmaceutical equivalence was demonstrated in 21 generics with undistinguishable slopes and intercepts (P > 0.66). Potency estimates were 99.8 to 100.5, 99.7 to 100.2 and 98.5 to 99.9% for generic products of amikacin, gentamicin and vancomycin, respectively.

**Conclusion:**

The proposed method allows rapid, cost-saving, precise, and accurate determination of pharmaceutical equivalence of drugs in pharmaceutical dosage-form, and may be used as a technique for testing generic antibiotics prior to their approval for human use.

## Background

Although resistance is a real and growing problem, the antimicrobials remain one of the three most prescribed drugs. Currently, the global anti-infective market is valued at US$66.5 billion with antibacterial agents accounting for over 50% of sales [1]. This remarkable cost has resulted in a massive use of generic drugs trying to assure unlimited access to cheap treatments, to the point that currently over half prescriptions include at least one generic product [2,3]. However, the phenomenal growth of generic drugs has brought concerns about their safety and efficacy because, opposite to innovators, generics are not required to demonstrate therapeutic efficacy [4-11]. In fact, pharmaceutical equivalence, defined as having equivalent amounts of the same active pharmaceutical ingredient (API) of the innovator, in the same dosage form, and produced under good manufacturing practices, constitutes the only criterion required for therapeutic equivalence of intravenous drugs [12]. Therefore, the quantification of API in standard samples or biological fluids is critical for drug regulatory agencies around the world.

Several analytical methods have been reported to quantify the concentration of API. They can be classified as microbiological assays (bioassay) [13,14], automated physicochemical assays (i.e. high performance liquid chromatography, HPLC) [15], and immunological assays (i.e. fluorescence polarization immunoassay) [16-18]. Automated chemical methods have largely replaced the microbiological assays to determine the antibiotic concentrations in body fluids (e.g. serum, plasma or urine), especially for therapeutic drug monitoring (TDM) in clinical settings, because they are more attractive than the classical bioassay in terms of speed, accuracy and precision [13,15,19]. However, automated assays and bioassays are frequently used and accepted by generic manufacturers as interchangeable and complementary to the other methods [20,21]. The main disadvantage of microbiological assays is the inability to quantify substances other than the API in the same matrix (i.e. metabolites or impurities). Although pharmaceutical-grade drugs usually are a mix of API plus preservatives, vehicles and impurities with different sources between manufacturers, regulatory agencies do not require the determination of these substances to define a generic as pharmaceutical equivalent, reducing the importance of this disadvantage [22].

The microbiological assay for antibiotics dates back to the demonstration of the lysozyme activity in an agar diffusion assay by Fleming, shortly followed by the agar diffusion assay for penicillin devised by Heatley [19]. Ever since, the main uses of agar diffusion assay were determination of potency of growth-inhibiting (i.e. antibiotics) and growth-promoting substances (i.e. amino acids) in blood, urine, and other body fluids and tissues, mainly for pharmacokinetic studies. An additional use of bioassay was recognized after the Drug Price Competition and Patent Term Restoration Act of 1984, when the measurement of antibiotic concentrations in various fluids was prominent to establish pharmaceutical equivalence of new generics and as manufacture quality control [13,23]. For this purpose, the calculation is based on the assumption of two symmetrical (each preparation has the same number of dose levels in the same ratio to one another) straight parallel-lines (so called "parallel-line assay"), one being a plot of the response (mean zone size) against the logarithmic concentration of the standard, the other being a plot of responses against the log concentration of the unknown product. The relative potency of unknown to standard reference is derived from the horizontal distance between the two lines. Usually, the recommended standard is a pure-grade product of known potency. However, no statistical procedure has been standardized to compare simultaneously the potency of two pharmaceutical-grade products (generic versus innovator) and the currently used method does not allow separation of concentration from potency. Additionally, complex pharmacopoeial guidelines on bioassay design and validation are available, including requirements for validity of parallel-line assays concerning precision, accuracy, sensitivity, and variance ratios for linear regression, non-parallelism and non-linearity. Here we propose a new and more understandable interpretation of the parallel-line assay results to determine pharmaceutical equivalence, comparing the dose-response relationship obtained with the pharmaceutical form of innovator versus generic by curve fitting analysis and performing an intra-house validation of the procedure assessing the same criteria recommended by international pharmacopoeias.

## Methods

### Antibiotics

Intravenous antibiotics for human use were bought from reputable local drugstores as needed. All products had been properly licensed by the drug regulatory agency of Colombia. We used the innovators of amikacin (Amikin^®^, Bristol-Myers Squibb, Guayaquil, Ecuador), gentamicin (Garamicina^®^, Schering-Plough SA, Bogota, Colombia) and vancomycin (Vancocin CP^®^, Eli Lilly & Compañia de Mexico SA de CV, Mexico), as well as the available generic products: four of amikacin, fifteen of gentamicin and three of vancomycin (N = 22 generic products). All products were reconstituted with calibrated micropipettes (Transferpette^®^, BRAND, Wertheim, Germany) following the manufacturer instructions for clinical use. Pure-grade reference powders of both aminoglycosides (Sigma Aldrich, St Louis, MO) and vancomycin (United States Pharmacopeia, Rockville, MD) were used as controls. The former were weighted in an analytical balance (Sartorius, Goettingen, Germany) and the latter was diluted to a final concentration of 50 mg/mL in distilled water.

### Media, strains and inocula

Testing strains were *Bacillus subtilis *ATCC 6633 for amikacin (and some experiments with vancomycin), *Staphylococcus aureus *ATCC 6538p for vancomycin, and *Staphylococcus epidermidis *ATCC 12228 for gentamicin. Seeding agar were Difco™ Antibiotic Media (Becton Dickinson & Co, Franklin Lakes, NJ) numbers 5 (amikacin), 8 (vancomycin) and 11 (gentamicin) [24].

To grow *S. aureus *and *S. epidermidis*, we followed CLSI protocols [25]. Briefly, each bacterial stock previously stored and frozen at -70°C was resuscitated on solid medium (in two successive Trypticase Soy Agar plates, Becton Dickinson, Sparks, MD) and five colonies were selected and passed to 10 mL of Trypticase Soy Broth (five 16 × 125 glass tubes labeled 1 to 5 with successive 1:10 dilutions). After overnight incubation at 37°C, we made a second transfer from the last tube with visible growth into 10 mL of fresh liquid medium (three 16 × 125 glass tubes labeled 6 to 8 with successive 1:10 dilutions). This second set was grown to attain an OD_580 nm _= 0.300 for *S. aureus *and 0.450 for *S. epidermidis *(Spectro 22^®^, Labomed Inc., Culver City, CA, USA), equivalent to a log-phase culture with approximately ~10^8 ^CFU/mL. We ruled out significant impact of *S. aureus' *cell clusters [26] by multiple standardizations of growth curves correlating OD with CFU count (data not shown). For *B. subtilis*, a spore suspension was prepared by growing the microorganism for one week at 37°C in a large bottle on Difco™ Antibiotic Media number 1. Then, the spores were suspended in sterile distilled water and heated for 30 minutes at 65°C, washed three times, and reheated at 65°C for 30 minutes before re-suspension in sterile distilled water. The final suspension containing ~10^8 ^CFU/mL was temporarily maintained at 15°C prior to inoculation of the bioassay agar.

### Device and pouring of glass-plates assay

A 36 × 36 cm glass plate previously described was modified to allow simultaneous runs in duplicates of all assays of the different generic products and innovator [27]. The device was routinely cleaned (iodine soap and water), disinfected (70% ethanol) and sterilized (steam autoclaving at 121°C). The seeding agar (3 mm of depth) was prepared following the manufacturer instructions, dispensed in a sterile 300 mL flask, and placed into a water bath (50°C) to maintain the agar in liquid state until poured. We inoculated the bioassay agar adding 2 mL of log-phase suspension of *S. aureus *or *S. epidermidis *in 100 mL of the corresponding antibiotic media or adding 3 mL of *B. subtilis *spore suspension for each 100 mL of antibiotic medium. These inoculum sizes (~2 × 10^6 ^CFU/mL) ensured sharply defined zone edges and a good slope steepness of the log dose-response line.

The solutions were applied in a simple sequential fashion down the columns, as described by Bennett et al. [27]. Incubation was carried out at 35°C for 18–22 h depending on the bacteria-agar combination, according with the instructions of the antibiotic media manufacturer [24]. The same researcher measured the zone sizes in all assays using an electronic caliper (Mitutoyo Corp., Kawasaki, Japan).

The use of mouse serum was approved, as well as the complete protocol, by the University of Antioquia Animal Experimentation Ethics Committee.

### Statistical Analyses

We determined the linearity, limit of quantification, precision, accuracy, and specificity to validate the method for testing pharmaceutical equivalence. For this purpose, the log-transformed concentrations (x-axis, log_10 _mg/L) of each product were plotted against their respective inhibition zone sizes (y-axis, mean diameter in mm); intercept and slope of the best straight line were obtained fitting the data to a linear model (expressed by the equation y = b + mx, where b is the y-intercept and m is the slope) by least-squares regression using SigmaPlot 9.0 (SPSS Inc., Chicago, IL). The concentrations used ranged from 0.5 to 256, 0.125 to 64, and 0.25 to 128 mg/L for amikacin, gentamicin and vancomycin, respectively. The goodness of fit to the model (linearity) was expressed as coefficient of determination (r^2^) and standard error of estimate (S_yx_). We also calculated the x-intercept (log_10 _mg/L) and slope of the regression line with 95% confidence intervals (95% CI) [28]. The regression approach to analysis of variance was used to determine the statistical significance of intercept and slope. Normality and constant variance assumptions were checked using Kolmogorov-Smirnov and Levene's tests, respectively (SPSS 15.0, SPSS Inc., Chicago, IL). In case of non-equal variance, we applied Welch's test to confirm the significance of the parameters derived from the linear model [29].

Using a symmetrical parallel-line assay we tested the pharmaceutical equivalence comparing slope and intercept of each generic product with those of the innovator by Overall Test for Coincidence of the Regression Lines, a statistical technique for Curve Fitting Analysis (CFA) (Prism 5.0, GraphPad Software, Inc., San Diego, CA) [30]. We defined potency as the slope of the linear regression and concentration as the anti-log of the x-intercept when y = 0. We also estimated the relative concentration when y is at the mid-point of the linear regression, because this is the point of minimal variation using the 95% confidence interval of the predicted line. Assuming that generic and innovator are the same product, a pharmaceutically equivalent generic must display a parallel and overlaid curve with respect to the innovator (P > 0.05 by CFA). On the other hand, parallel curves with different intercepts meant identical APIs but at significantly higher or lower concentration, while lack of parallelism implied that different products were being tested, without further considerations. We also calculated the relative potency of each generic to innovator as the x-distance between the two lines, as recommended by the international pharmacopoeias [20].

The limit of quantification was defined visually as the smallest amount of drug that still produced a clearly distinguishable inhibition zone around the diameter of an empty well (3.5 mm). The limit of detection was calculated using the standard deviation and slope method recommended by ICH guidelines.

The repeatability of the assay was determined using a minimum of three concentrations of each antibiotic by triplicates during the same day or under similar experimental conditions but with different biological matrices (water instead of serum to dilute samples) and plates (intra-assay precision), and comparing the results of assays on different days (inter-day precision). These were expressed as means with standard deviations and coefficients of variation (CV) [31].

To test the ability of the assay to detect significant differences in concentration of the API (accuracy), we prepared a 5-point standard curve (two-fold dilutions from 128 to 8 mg/L) using vancomycin reference powder (USP) and compared it with samples containing 10, 15 and 20% more vancomycin. Briefly, a vial containing 100.5 mg of the antibiotic was dissolved in 2.01 mL of distilled water to a final concentration of 50 mg/mL and serially diluted to achieve 1 mL at 128 mg/L. Then, 0.1 mL aliquots of this solution were diluted to obtain a set of three extra concentration groups over three selected points in the standard curve (16, 32 and 64 mg/L): Group I with 10% more (17.6, 35.2 and 70.4 mg/L), Group II with 15% more (18.4, 36.8 and 73.6 mg/L) and Group III with 20% more (19.2, 38.4 and 76.8 mg/L) vancomycin. Intercepts and slopes of linear regressions produced by the reference standard and each extra group were compared by CFA [32].

To show the ability of the microbiological assay to unambiguously assess the API in presence of all other expected components in a pharmaceutical-grade product for clinical use (specificity), it was necessary to subject the innovator of vancomycin to specific stress conditions to increase degradation subproducts. In this case, we compared by CFA the intercept and slope of a freshly prepared standard curve (16 to 256 mg/L) against those obtained after 2, 4, 8 and 16 hours of heating at 80°C.

## Results

### Antibiotics

Table 1 shows detailed information for all amikacin (AMK), gentamicin (GNT) and vancomycin (VAN) products studied. All reference powders (100%), 1 of 4 (25%), 2 of 15 (13%), and 3 of 3 (100%) generics of AMK, GNT and VAN, respectively, were manufactured outside Colombia and legally imported for clinical use. It should be noted that one company (Vitrofarma SA, Bogota, Colombia) manufactured 5 of 15 (33%) generics of GNT.

**Table 1:** Source and batch of antibiotics included in the study

**Product Code**	**Batch**	**Manufacturer**	**Distributor**
AMK-BMS (innovator)	01J115	Bristol-Myers Squibb (BMS), Guayaquil, Ecuador	Bristol-Myers Squibb, Cali, Colombia

AMK-Carlon	278V0704	Carlon Ltda, Bogota, Colombia	Item

AMK-Gencol	0200	Laboratorios Chalver, Bogota, Colombia	Genericos de Colombia (Gencol), Bogota, Colombia

AMK-Pisa	121859	Laboratorios Pisa SA de CV, Guadalajara, Mexico	Laboratorios ECAR Ltda, Medellin, Colombia

AMK-Scalpi	AK010348	Consorcio Farmionni-Lubelca, Bogota, Colombia	Farmionni Scalpi SA, Bogota, Colombia

AMK-Sigma (Reference Powder)	120K1643	Sigma Chemical Co, St Louis, MO, USA	Not applicable

GNT-SP (innovator)	CB3AMKB04	Schering-Plough SA (SP), Bogota, Colombia	Item

GNT-Abbott	75-024-DK	Abbott Laboratories, North Chicago, IL, USA	Not available

GNT-Az pharma	303030	Vitrofarma SA, Bogota DC, Colombia	AZ-Pharma SA, Bogota, Colombia

GNT-Biochemie	07102321	Biochemie GmbH, Kundl, Austria	Novartis de Colombia SA, Bogota, Colombia

GNT-Biogenta	0402	Laboratorios Chalver, Bogotá, Colombia	Chalver Farmaceutica (Biogenta), Bogota Colombia

GNT-Colmed	01005	Colmed Internacional, Barranquilla, Colombia	Procaps SA, Barranquilla, Colombia

GNT-Genfar	030703	Viteco SA, Bogota, Colombia	Lab. Genericos Farmaceuticos, Bogota, Colombia

GNT-Lab America	0980303	Arbofarma SA, Bogota, Colombia	Laboratorios America SA, Medellin, Colombia

GNT-Labinco	01013C	Laboratorios Ryan, Bogota, Colombia	Laboratorio Internacional (Labinco), Bogota, Colombia

GNT-La Sante	0310	Viteco SA, Bogota, Colombia	Laboratorios La Sante, Bogota, Colombia

GNT-Memphis	2208I101	Vitrofarma SA, Bogota, Colombia	Memphis products SA, Bogota, Colombia

GNT-MK	3P066	Vitrofarma SA, Bogota, Colombia	Tecnoquimica SA (MK), Cali Colombia

GNT-Ophalac	004013	Vitrofarma SA, Bogota, Colombia	Laboratorios Farmaceuticos Ophalac, Bogota, Colombia

GNT-Pentacoop	33544	Laboratorios Ryan, Bogota, Colombia	Pentacoop SA, Bogota DC, Colombia

GNT-Recipe	301094	Vitrofarma SA, Bogota, Colombia	Linea Recipe^® ^of Laboratorios Bussié SA, Colombia

GNT-Scalpi	GE020619	Consorcio Farmionni-Lubelca, Bogota, Colombia	Farmionni Scalpi SA, Bogota, Colombia

GNT-Sigma (Reference Powder)	10k1510	Sigma Chemical Co, St Louis, MO, USA	Not applicable

VAN-Lilly (Innovator)	A014744	Eli Lilly & Compañia de Mexico, Mexico	Eli Lilly Interamericana Inc., Bogota, Colombia

VAN-Abbott	03703Z7	Abbott Laboratories, Chicago, IL, USA	Abbott Laboratories de Colombia, Bogota, Colombia

VAN-APP	120740	American Pharmaceutical Partners (APP), LA, USA	Comedica Ltda., Bogota, Colombia

VAN-Proclin	6679	Laboratorios Northia S.A.C.I.F.I.A, Argentina	Proclin Pharma SA, Bogota, Colombia

VAN-USP	70900L	United States Pharmacopeia (USP), Rockville, MD, USA	Not applicable

### Goodness of fit of the linear regression model to data

**Figure 1:**
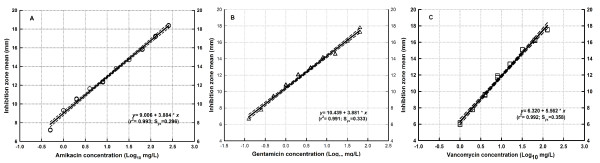
**Linear regression of the innovator products**. Linear regression of the concentration-effect relationship of the innovator products of amikacin (A), gentamicin (B) and vancomycin (C) showing minimal scatter and excellent fit (r^2 ^≥ 0.993) of the data to the model.

Figure 1 shows the log concentration-response relationship and the best straight line predicted from data obtained by microbiological assay for the innovator of amikacin (A), gentamicin (B) and vancomycin (C). All cases exhibited a linear relationship between the logarithm of the concentration (log_10 _mg/L) and the diameter (mm) of the zones of inhibition with high coefficients of determination (r^2 ^≥ 0.991), low standard errors of the estimate (S_yx _≤ 0.358) and statistically significant intercept and slope (P < 0.001 by ANOVA). Almost all products passed normality and constant variance tests, except for some amikacin generics that failed the Levene's test. Welch-ANOVA confirmed the significance of parameters derived from the linear regression for these products (P < 0.0001).

### Determination of pharmaceutical equivalence

**Figure 2:**
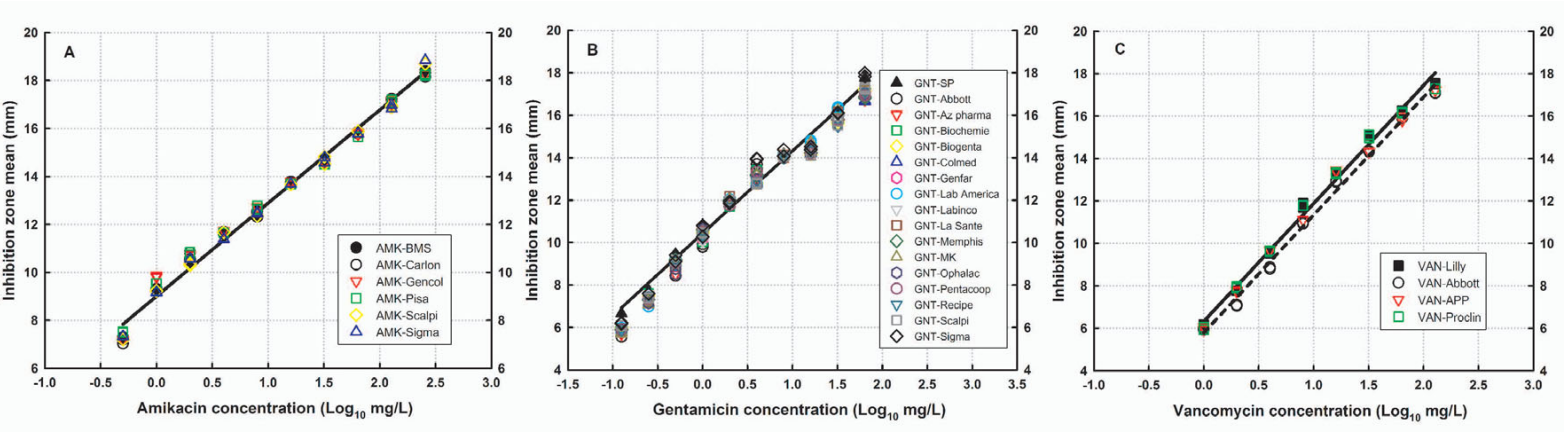
**Multiple linear regression for all generics and their respective innovators**. Except for VAN-Abbott (black circles on panel C), data of concentration-effect relationship of all generic products are overlaid and can be described by the simple regression (black line) of the innovator product of amikacin (A), gentamicin (B) and vancomycin (C). Significant difference in the intercept of VAN-Abbott is evident by its linear regression behavior (black short dash line on panel C) compared with the regression of the innovator.

Except for one generic product (VAN-Abbott) that exhibited a different intercept (P < 0.008 by CFA), the log concentration-response relationships of all generic products and reference powders were parallel and overlaid to their respective innovator linear curve without significant difference by CFA (P ≥ 0.656, Table 2), demonstrating that all products had the same biologic activity (potency) and concentration (intercept). The high goodness of fit of the model allowed the use of innovator product equations as common linear equations for all generics (Figure 2, panels A to C) to predict the concentration (mg/L) of a sample (generic product) from an inhibition zone (mm).

**Table 2:** Potency estimate and parameters derived from linear regression with their statistical comparison of generics versus innovator by Curve Fitting Analysis

**Product**	**r**^2^	**Intercept [95% CI]**	**P-value**	**Slope [95% CI]**	**P-value**	**Potency Estimate (%)**
AMK-BMS	0.99	9.006 [8.646 to 9.366]	0.8799	3.884 [3.619 to 4.148]	0.6559	100.00

AMK-Carlon	0.99	9.082 [8.651 to 9.514]		3.789 [3.472 to 4.105]		99.83

AMK-Gencol	0.99	9.238 [8.738 to 9.737]		3.731 [3.365 to 4.098]		100.51

AMK-Pisa	0.99	9.244 [8.860 to 9.629]		3.698 [3.416 to 3.980]		100.31

AMK-Scalpi	0.99	8.920 [8.552 to 9.287]		3.935 [3.665 to 4.204]		99.77

AMK-Sigma	0.99	8.945 [8.575 to 9.315]		3.926 [3.655 to 4.198]		99.89

GNT-SP	0.99	10.44 [10.16 to 10.720]	0.9472	3.881 [3.594 to 4.167]	0.9984	100.00

GNT-Abbott	0.98	10.00 [9.557 to 10.440]		4.150 [3.695 to 4.605]		99.72

GNT-Az pharma	0.98	10.02 [9.576 to 10.460]		4.021 [3.570 to 4.473]		99.68

GNT-Biochemie	0.98	10.08 [9.668 to 10.500]		4.038 [3.613 to 4.463]		99.75

GNT-Biogenta	0.98	10.13 [9.736 to 10.510]		3.925 [3.526 to 4.324]		99.74

GNT-Colmed	0.98	10.13 [9.701 to 10.550]		3.918 [3.483 to 4.354]		99.74

GNT-Genfar	0.99	10.04 [9.697 to 10.380]		4.091 [3.738 to 4.443]		99.73

GNT-Lab America	0.99	10.09 [9.708 to 10.470]		4.143 [3.754 to 4.532]		99.79

GNT-Labinco	0.99	10.11 [9.749 to 10.470]		3.994 [3.623 to 4.366]		99.76

GNT-La Sante	0.98	10.11 [9.668 to 10.550]		4.106 [3.655 to 4.558]		99.80

GNT-Memphis	0.98	10.15 [9.694 to 10.610]		3.935 [3.465 to 4.406]		99.77

GNT-MK	0.98	10.09 [9.672 to 10.510]		4.146 [3.715 to 4.577]		99.68

GNT-Ophalac	0.98	10.02 [9.571 to 10.460]		4.006 [3.549 to 4.463]		99.68

GNT-Pentacoop	0.98	10.13 [9.690 to 10.560]		3.962 [3.514 to 4.409]		99.76

GNT-Recipe	0.98	10.10 [9.700 to 10.510]		3.926 [3.512 to 4.340]		99.72

GNT-Scalpi	0.98	10.13 [9.720 to 10.540]		3.919 [3.501 to 4.337]		99.74

GNT-Sigma	0.98	10.36 [9.889 to 10.830]		4.109 [3.629 to 4.590]		100.02

VAN-Lilly	0.99	6.320 [5.715 to 6.924]	0.0201	5.562 [5.082 to 6.042]	0.8594	100.00

VAN-Abbott	0.99	5.782 [5.248 to 6.316]		5.558 [5.134 to 5.982]		98.51

VAN-APP	0.99	6.281 [5.734 to 6.828]		5.359 [4.925 to 5.793]		99.30

VAN-Proclin	0.99	6.388 [5.721 to 7.054]		5.462 [4.933 to 5.991]		99.90

**Figure 3:**
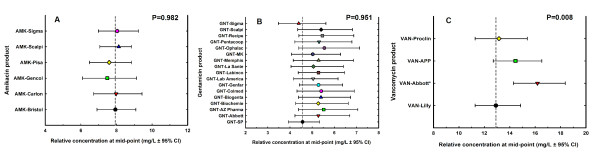
**Pharmaceutical equivalence determined by comparison of the relative potency of the generic products against the innovator**. Relative potency at the linear regression midpoint (mean and 95% CI) of the innovator and generic products of amikacin (2A), gentamicin (2B) and vancomycin (2C). Except for vancomycin Abbott, there were no significant differences in potency between generics and their corresponding innovator, confirming pharmaceutical equivalence.

The potency estimate of generic products ranged from 99.77 to 100.5, 99.68 to 100.2 and 98.51 to 99.9% relative to the innovator of amikacin, gentamicin and vancomycin, respectively (Table 2). In the same order, the comparison of relative concentrations of all generic products at the mid-point of their linear regression against the innovator by confidence intervals did not show significant differences (Figure 3, panels A to C), except for VAN-Abbott that displayed the same potency and 25% greater concentration of API (16.2 ± 1.05 vs. 12.9 ± 1.06 mg/L, P = 0.001).

### Other validation parameters

**Figure 4:**
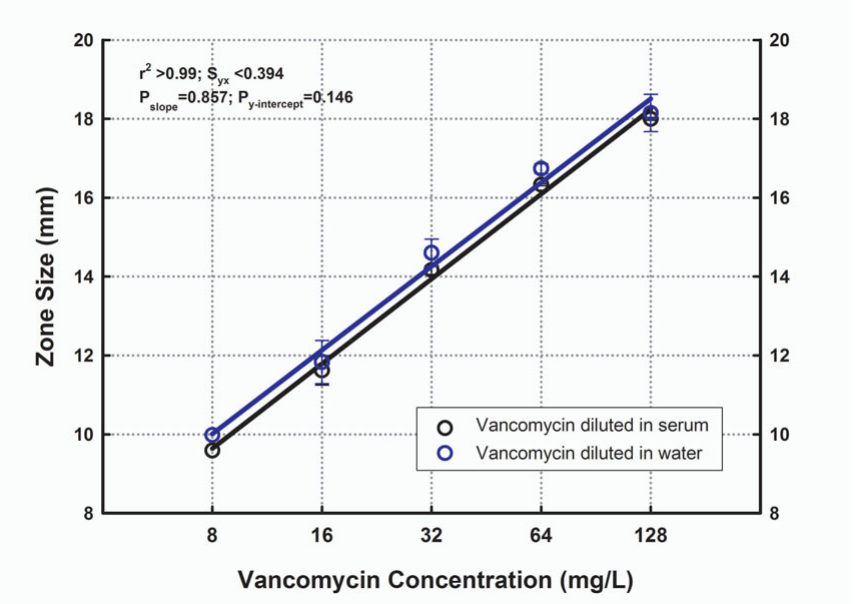
**Linear regression of the concentration-effect relationship of vancomycin diluted in different media (water and murine serum)**. There was no statistically significant difference between the two data sets, showing that all points fit better to a single line, and the assay is not affected by changes in the biological matrix.

The calculated limits of quantification were 0.13, 0.10 and 0.15 mg/L for amikacin, gentamicin and vancomycin, respectively. Table 3 shows the results of repeatability in a five-concentration assay (8 to 128 mg/L) for the reference powder of vancomycin performed the same day using different plates (intra-assay variation), and working on different days (inter-day variation). The CV ranged from 4.6% to 11.0% within the same day, and from 1.0% to 10.3% between days, respectively. Wilcoxon signed-rank test did not show significant differences between the results of similar experiments made in different days (P = 0.426). The variation of the bioassay was also assessed using different biological matrices (mouse serum vs. distilled water) as diluents (Figure 4). There were no statistical differences comparing the linear regression of each matrix by CFA (P_*y*-*intercept *_= 0.311 and P_*slope *_= 0.857).

**Table 3:** Precision of the vancomycin bioassay

**Vancomycin concentration (mg/L)**	**Inhibition zone diameters (mean in mm ± SD)**	**Coefficient of variation (%) Intra-day**	**Coefficient of variation (%) Inter-day**
128	18.00 ± 0.04	8.3	10.3

64	16.33 ± 0.02	5.6	10.5

32	14.16 ± 0.11	11.0	1.0

16	11.62 ± 0.37	7.4*	2.3

8	9.59 ± 0.00	4.6	6.5

*An outlier value was excluded from the calculations (we only used five data to compute the CV)

**Figure 5:**
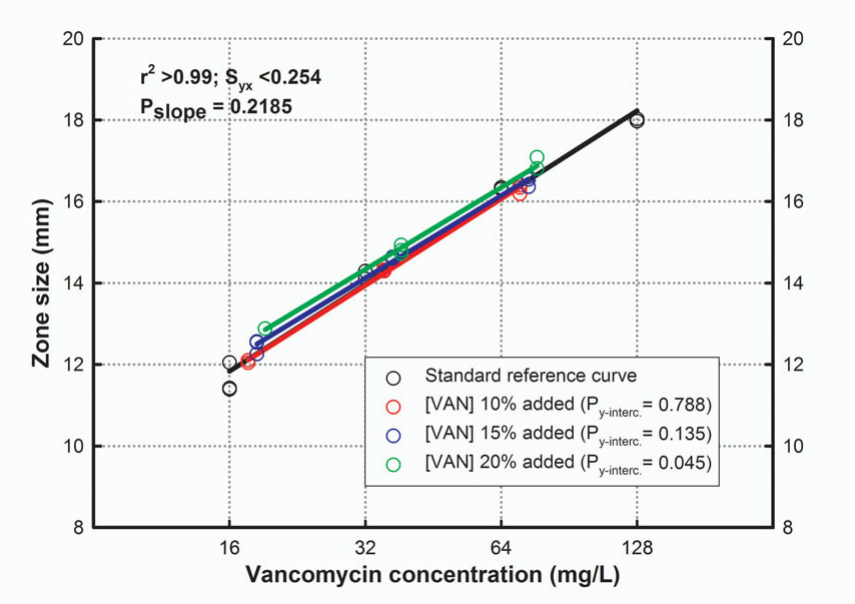
**Comparison of a standard curve of innovator vancomycin with samples containing 10% (red), 15% (blue) and 20% (green) more antibiotic**. The curve fitting analysis showed no difference in slopes (i.e. all samples had the same biological activity) but significantly different intercept (i.e. more concentration) in the 20% group (indicating the threshold for statistical significance of the assay).

Figure 5 shows the accuracy of the assay to detect changes in concentration of the same API. Whereas the CFA comparison of the slopes produced by the standard and extra-vancomycin concentration groups did not show differences (P = 0.219), we found a significant difference (as expected) in the y-intercepts (P = 0.0002). These results corroborate that our method can discriminate changes of concentration (intercept) even for API with identical biologic activity (slopes). To define the group responsible for the observed y-intercept difference, we used an Overall Test for Coincidence of Two Linear Regressions (each group versus standard), which only showed a significant difference between the standard vancomycin curve and the 20% extra-concentration group (P = 0.045).

**Figure 6:**
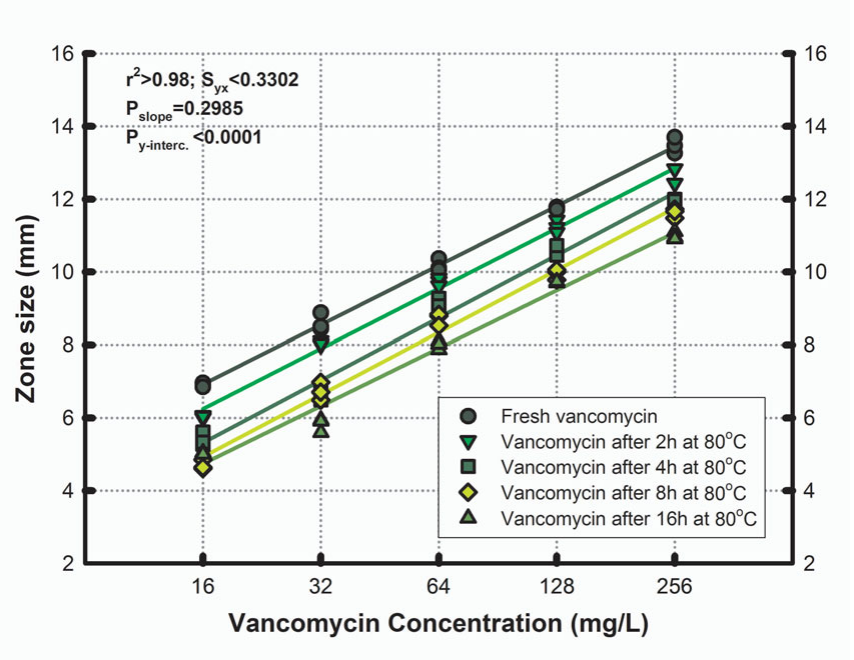
**Effect of heating at 80°C on vancomycin samples over a 16-hour period**. Compared with the fresh product, the concentration of active principle gradually declines (as shown by different intercepts). The identical slopes indicate that the assay only measures the vancomycin active principle and that degradation products lack significant biological activity.

Figure 6 shows the impact on the linear regression of heating vancomycin (16 to 256 mg/L) at 80°C for 2, 4, 8 and 16 hours compared against that produced by a standard curve of fresh vancomycin (at room temperature). Independently of heating time, significant changes on API concentration (P_*y*-*intercept *_< 0.0001) could be detected by the method without evidence of modification on the biological activity (P_*slope *_= 0.2985). Compared with fresh vancomycin, the inhibition zones diminished proportionally to the heating time and the curves were below the control at room temperature. The percentage of recovery of active ingredient of vancomycin ranged from 69 to 83, 44 to 64, 39 to 54 and 31 to 44% after 2, 4, 8 and 16 hours, respectively.

## Discussion

The microbiological assay has been used extensively to estimate the potency of growth-inhibiting substances by comparing their quantitative effect with a reference standard of defined potency [14,33,34]. Here, we developed a different application aimed to compare with a gold standard (innovator) the concentration and potency of pharmaceutical-grade generic products for intravenous use. To our knowledge, it is the first time that the bioassay is validated to determine pharmaceutical equivalence of generic intravenous antibiotics.

It is a common belief that, being a biological assay, the agar-diffusion assay is subject to biological error and therefore less reliable than physicochemical methods (e.g. HPLC) [13]. However, different to the macrobiological assay that uses a limited number of higher organisms (usually animals) with a natural variability, the microbiological assay employs tens of millions of individuals (bacteria) as biological indicators that usually react uniformly to the active substance tested. Therefore, the biological error is not the usual source of error in microbiological assays, allowing meaningful results with a precision similar to any chemical or physicochemical method [16,33,35]. In fact, we observed an excellent goodness of fit of the linear regression model to data (r^2 ^≥ 0.99; S_yx _≤ 0.358) with highly precise results and minimal intraday and inter-day variation (Table 3), which is very close to physicochemical methods (~8%), and with good accuracy and sensitivity permitting to detect significant differences on API concentration either by excess (Figure 5) or defect (Figure 6). Furthermore, our method widely exceeds the linearity, precision and accuracy requirements for validation of the international pharmacopoeias (i.e. FDA) for physicochemical methods [20,36]. Considering that pharmaceutical equivalence implies to prove that the API concentration in a generic product is located within an accepted range respect to a gold standard (80–120%), precision and accuracy of the method are the most important criteria to be considered. Bearing in mind that we compared active ingredients and not pharmaceutical impurities, our results suggest that the bioassay is as good as physicochemical methods to correctly determine pharmaceutical equivalence of biologic products (antibiotics) [37].

The mathematical principles employed to compare generic and innovators are simple and derived from the well-known guidelines for parallel-line assays established by regulatory agencies [13,14,38,39]. Here, we employed curve fitting analysis to compare the linear regressions of generics against the innovator and defined concentration and potency using the same parameters (y-intercept and slope, respectively) of the linear equation. Logically, if a generic drug is identical to the innovator, then their standard curves (intercept and slope) from a bioassay should not differ significantly (pharmaceutical equivalents).

As we confirmed for vancomycin, adding or subtracting active principle to the same product induces a corresponding shift in the intercept of the concentration-response curve without affecting the slope (potency). The assay is sensitive to detect variations in concentration ≥ 120% (see 20% extra-concentration group in Figure 5) and ≤ 85% (see 2 hours post-incubation group in Figure 6). These values are similar to the variation range (80 to 120%) of API concentration accepted for many generic antibiotics [20].

As we expected, almost all generics approved by the drug regulatory agency in Colombia (except by VAN-Abbott) were identical to the innovator displaying equal slopes (potency) and intercepts (concentration) as demonstration of their pharmaceutical equivalence. It has not escaped our attention that the concentration and slope of all five gentamicin products produced by the same manufacturer were pharmaceutical equivalents and did not exhibit contradictory results (Table 2).

Vancomycin for clinical use is a mixture of active principle (factor B) and impurities (crystalline degradation products or CDP-1) that differ widely in inhibitory potency (factor B at least 1000-times superior to CDP-1) [40,41]. The exposure of innovator vancomycin to heat, previously described by Sheldrick et al [42,43], progressively induces the conversion of factor B to CDP-1, reducing the concentration of the active principle (intercept) without affecting the original potency (slope) of the product (Figure 6). However, the concentration of these degradation products cannot be measured, certainly a limitation for evaluating impure drugs (i.e. oral forms) or prodrugs (e.g. clindamycin phosphate) by our method.

As stated earlier, therapeutic equivalence of generic intravenous antibiotics is based solely on the demonstration of their pharmaceutical equivalence without further testing. Considering the critical importance of antiinfective chemotherapy in terms of disease control and emergence of resistance, it is necessary to test the assumption that pharmaceutical equivalent generics are also therapeutic equivalent to innovators. The methodology described here constitutes the first step in this direction prior to validate an animal model of infection or a clinical trial to test in vivo efficacy and correlate it with the microbiological assay results.

## Conclusion

The microbiological assay demonstrated to be a precise, reliable and suitable method to determine pharmaceutical equivalence of intravenous antibiotics by comparison of their concentration and potency.

## Abbreviations

AMK: Amikacin; API: Active Pharmaceutical Ingredient; CFA: Curve Fitting Analysis; GNT: Gentamicin; ICH: International Conference on Harmonisation; r^2^: Coefficient of Determination; S_yx_: Standard Error of Estimate; TDM: Therapeutic Drug Monitoring; 95% CI: 95% Confidence Interval; VAN: Vancomycin

## Competing interests

Zuluaga has received honoraries for lectures from Pfizer and Roche, and received financial support from Pfizer, AztraZeneca and Merck Sharp & Dohme (MSD) to participate in scientific international meetings. Rodriguez received financial support to participate in international meetings from AztraZeneca and Wyeth. Agudelo received financial support to participate in international meetings from Wyeth and Bristol-Myers Squibb (BMS). Vesga has received honoraries for lectures and financial support to participate in international meetings from AstraZeneca, GlaxoSmithKline, Pfizer, BMS, MSD, Sanofi-Aventis and Wyeth, and has been a member of advisory boards for Wyeth. None of these or any other pharmaceutical company supported the present study.

## Authors' contributions

AFZ contributed during the experimental process, performed the analysis and interpretation of data and drafted the manuscript. MA and CAR carried out the experiments and contributed during the analysis of data and manuscript preparation. OV conceived the study, obtained funding, designed and directed the execution and analysis of data, edited the manuscript, and gave final approval for its publication.

## Pre-publication history

The pre-publication history for this paper can be accessed here:


